# Supplementary material for: Affective certainty and congruency of touch modulate the experience of the rubber hand illusion
Source: Sci Rep. 2019 Feb 22;9:2635. doi: 10.1038/s41598-019-38880-5 (PMC6385173; doi:10.1038/s41598-019-38880-5)
Supplement: Supplementary file 1 — Supplementary Material [file 41598_2019_38880_MOESM1_ESM.docx]

**Affective certainty and congruency of touch modulate the experience of the rubber hand illusion**

Maria Laura Filippetti^1,2^, Louise P. Kirsch^2^, Laura Crucianelli^2^, Aikaterini Fotopoulou^2^

^1^ Centre for Brain Science, Department of Psychology, University of Essex, CO4 3SQ, Colchester, UK

^2^ Research Department of Clinical, Educational & Health Psychology, University College London, WC1E 7HB, London UK

**Supplementary Material**

These data provide essential information with regard to 1) the selection of fabrics for our subsequent studies (pilot data) and 2) tactile and visual discrimination baseline values, thus ensuring the presence of baseline discrimination of our selected materials in terms of pleasantness and unpleasantness.

**Pilot data**

*Figure 1S These bar graphs show pleasantness (top graph) and unpleasantness (bottom graph) ratings of 12 selected materials with different degrees of pleasantness, contrasted in a visual only, tactile only, and visuo-tactile conditions. For our main study, we selected the ‘velcro hooks’ and ‘synthetic wool’ fabrics based on these data and visual matching of size and colour.*

**Tactile and visual discrimination baseline measure**

|  | EXPERIMENT 1 | | | | EXPERIMENT 2 | | | |
| --- | --- | --- | --- | --- | --- | --- | --- | --- |
| Valence rating | **Tactile test** | | **Visual test** | | **Tactile test** | | **Visual test** | |
|  | Wool | Velcro | Wool | Velcro | Wool | Velcro | Wool | Velcro |
| Pleasantness | 74.31 (19.29) | 38.08 (20.16) | 79.03 (18.36) | 22.64 (21.54) | 76.94 (21.72) | 37.04 (25.76) | 77.37 (21.74) | 26.43 (27.31) |
| Unpleasantness | 18.92 (22.93) | 45.44 (17.14) | 13.28 (24.41) | 71.61 (25.76) | 11.76 (23.15) | 47.39 (22.56) | 14.86 (27.56) | 67.90 (27.56) |
| Arousal | 55.19 (22.20) | 47.42 (26.52) | 58.31 (24.53) | 46.86 (36.58) | 52.24 (23.42) | 40.47 (28.98) | 57 (26.51) | 38.27 (28.85) |

*Table 1S. Mean and standard deviation (in parenthesis) of the subjective valence ratings (pleasantness, unpleasantness, arousal) for the visual and tactile tests in Experiment 1 and 2.*

For Experiment 1, we found a negative correlation between pleasantness and unpleasantness ratings on the tactile baseline test for both pleasant (synthetic wool, r = -0.608, p < 0.001) and non-pleasant (Velcro, r = -0.578, p < 0.001) fabrics. Similarly, we found a negative correlation between pleasantness and unpleasantness ratings on the visual baseline test for both pleasant (synthetic wool, r = -0.451, p = 0.006) and non-pleasant (Velcro, r = -0.786, p < 0.001) fabrics. We found similar results for Experiment 2. For the tactile baseline test, we found a negative correlation between pleasantness and unpleasantness ratings for both pleasant (synthetic wool, r = -0.575, p < 0.001) and non-pleasant (Velcro, r = -0.637, p < 0.001) fabrics. For the visual baseline test, we again found a negative correlation between pleasantness and unpleasantness ratings for both pleasant (synthetic wool, r = -0.899, p < 0.001) and non-pleasant (Velcro, r = -0.803, p < 0.001) fabrics. Overall, these correlation analyses show that the higher the pleasantness reported by the participant in relation to the visual or tactile experience of the fabric, the lower its unpleasantness.

**Subcomponent analysis of embodiment questionnaire**

We analyzed the questionnaire data, by taking into account its three embodiment sub-components (ownership, location and agency – Longo et al., 2008) separately. For both experiments, results were in line with the composite embodiment score reported in the manuscript. Specifically, Wilcoxon signed rank tests revealed a main effect of Stroking, with synchronous stroking producing significantly higher ownership, location and agency component scores than asynchronous stroking (Experiment 1 - ownership: Z = 3.17, p = 0.002; location: Z = 2.51, p = 0.001; agency: Z = 3.09, p = 0.002; Experiment 2 – ownership: Z = 4.74, p < 0.001; location: Z = 4.45, p < 0.001; agency: Z = 4.30, p < 0.001). The Congruency manipulation was significant, with certainty/congruency of the fabric producing higher ownership, location and agency scores than incongruency of the fabric (Experiment 1 - ownership: Z = -3.12, p = 0.002; location: Z = -2.66, p = 0.008; agency: Z = -3.05, p = 0.002; Experiment 2 - ownership: Z = -2.65, p = 0.008; location: Z = -5.06, p < 0.001; agency: Z = -2.40, p = 0.017). The Fabric manipulation was not significant, with pleasant fabric producing similar ownership, location and agency scores than the less pleasant fabric (Experiment 1 - ownership: Z = -.83, p = 0.405; location: Z = 0.58, p = 0.561; agency: Z = -.86, p = 0.390; Experiment 2 - ownership: Z = -.85, p = 0.395; location: Z = 1.54, p = 0.125; agency: Z = -.61, p = 0.543).
